# Teaching a Robot Bimanual Hand-Clapping Games via Wrist-Worn IMUs

**DOI:** 10.3389/frobt.2018.00085

**Published:** 2018-07-17

**Authors:** Naomi T. Fitter, Katherine J. Kuchenbecker

**Affiliations:** ^1^Interaction Lab, Department of Computer Science, University of Southern California, Los Angeles, CA, United States; ^2^Haptics Group, Department of Mechanical Engineering and Applied Mechanics, University of Pennsylvania, Philadelphia, PA, United States; ^3^Haptic Intelligence Department, Max Planck Institute for Intelligent Systems, Stuttgart, Germany

**Keywords:** physical human-robot interaction, social robotics, motion classification, human-robot teaming, hand-clapping games

## Abstract

Colleagues often shake hands in greeting, friends connect through high fives, and children around the world rejoice in hand-clapping games. As robots become more common in everyday human life, they will have the opportunity to join in these social-physical interactions, but few current robots are intended to touch people in friendly ways. This article describes how we enabled a Baxter Research Robot to both teach and learn bimanual hand-clapping games with a human partner. Our system monitors the user's motions via a pair of inertial measurement units (IMUs) worn on the wrists. We recorded a labeled library of 10 common hand-clapping movements from 10 participants; this dataset was used to train an SVM classifier to automatically identify hand-clapping motions from previously unseen participants with a test-set classification accuracy of 97.0%. Baxter uses these sensors and this classifier to quickly identify the motions of its human gameplay partner, so that it can join in hand-clapping games. This system was evaluated by *N* = 24 naïve users in an experiment that involved learning sequences of eight motions from Baxter, teaching Baxter eight-motion game patterns, and completing a free interaction period. The motion classification accuracy in this less structured setting was 85.9%, primarily due to unexpected variations in motion timing. The quantitative task performance results and qualitative participant survey responses showed that learning games from Baxter was significantly easier than teaching games to Baxter, and that the teaching role caused users to consider more teamwork aspects of the gameplay. Over the course of the experiment, people felt more understood by Baxter and became more willing to follow the example of the robot. Users felt uniformly safe interacting with Baxter, and they expressed positive opinions of Baxter and reported fun interacting with the robot. Taken together, the results indicate that this robot achieved credible social-physical interaction with humans and that its ability to both lead and follow systematically changed the human partner's experience.

## Introduction

As robot use expands from independent operation in factories to cooperative responsibilities in environments like hospitals and schools, social skills become an increasingly important factor for robot developers to consider. Socially capable robots are known to be able to deliver better interaction experiences in everyday human-populated environments (Fong et al., 2003). Although direct physical contact between humans and robots introduces new safety requirements, mastering such interactions could increase a robot's ability to help people (Ikemoto et al., 2012) and promote the acceptance of robots by the general population.

Human children frequently engage in hand-clapping games (patterns of hand-to-hand contacts carried out by two people) for entertainment, to learn about others, and to make friends (Brodsky and Sulkin, 2011). Accordingly, as an initial foray into equipping robots with social-physical human-robot interaction (spHRI) skills, we chose to investigate human-robot hand-to-hand contact during playful hand-clapping games like “Pat-a-cake” and “Slide.” We prepared to run this study by connecting our past work on classifying human hand-clapping motions recorded via inertial sensors (Fitter and Kuchenbecker, 2016c) with our previously developed methods for making a robot clap hands in human-like ways (Fitter and Kuchenbecker, 2016b). The result of this union is sensor-mediated human-robot interaction (HRI) during which each participant (the human and the robot) physically mimics the movements of the other one at different times during the game.

After section Related Work presents related work, section Hand Motion Classification describes how we developed a capable system for repeatedly classifying human hand-clapping motions. Section Hand-Clapping Study Methods details our exploration and evaluation of a skilled Baxter robot that claps hands with people in various game modes. Sections Results and Discussion outline the results of this user study and discuss the findings and their implications for HRI.

## Related work

Our work sits at the intersection of social robotics and physical HRI (pHRI). The field of social robotics studies robots in social scenarios, usually without physical contact between the robot and the interacting humans (Fong et al., 2003). Within this field, the subtopic of socially assistive robotics leverages unique robot strengths in areas such as education and healthcare (Feil-Seifer and Mataric, 2005). In contrast, pHRI focuses more on interaction safety issues rather than social design (De Santis et al., 2008). pHRI might also be used to help a robot stay safe while navigating an unknown environment (Iwata and Sugano, 2005). Only a handful of pHRI investigations consider the social aspects of robotic contact. One previous study of how a human feels when touched by a robot in a medical setting found that people preferred procedural medical touch to compassionate pats from a robot (Chen et al., 2011). Experiments at this social-physical intersection, such as our work and the following related topics, elucidate how people perceive social-physical robots and how researchers can appropriately apply spHRI to aid people.

We are energized by prior research that combines social robotics and pHRI because touch is an essential pathway for human connection and emotion (Sonneveld and Schifferstein, 2008). In particular, physical interaction with the hands greatly aids human understanding and serves as a channel for complex sensation and expression (Klemmer et al., 2006). A few instances of spHRI appear in previous literature. The Haptic Creature Project, for example, explores an expressively actuated cat-sized furry robotic companion that responds to physical contact from humans (Yohanan and MacLean, 2008). Haptic feedback has also been leveraged to explore the subjective and objective results of physical human-robot collaboration in tasks such as joint target acquisition and object manipulation (Reed and Peshkin, 2008; Feth et al., 2011). In our spHRI work, the robot has a humanoid form and directly touches the human, rather than interacting through an external object.

Our research on bimanual hand-clapping robots additionally draws on the area of social motor coordination (also known as joint action). This topic is being actively explored not only in the HRI community, but also in research on human-human interaction (Schmidt et al., 2011). For example, one investigation proposes a video game that uses electrodermal activity-sensing controllers to detect hand-to-hand contacts between players for more enjoyable social gameplay (Baba et al., 2007). Similar research efforts by Kim et al. (2014) outline the design and testing of an electrodermal activity-sensing wrist-worn watch designed to increase intimacy in a workplace environment. In the HRI space, our initial inspiration for a jointly-acting hand-clapping robot was the popular PR2 demo entitled “Please do not touch the robot,” during which people can high five, fist bump, and hug the Willow Garage PR2 robot (Romano and Kuchenbecker, 2011).

Our social-physical Baxter robot is designed to use inertial measurement units (IMUs) to understand the hand motions of its human partner. Previous research has shown that motion classification using IMUs and other inertial sensing systems can be more efficient and accurate than processing of visual input. Past studies of body-mounted sensors for action recognition include motion prediction for full-body ambulatory behaviors from five IMUs (Altun and Barshan, 2010; Altun et al., 2010) and motion and gesture recognition from a complex system of IMUs and accelerometers (Chavarriaga et al., 2013). Almost all such work hinges on machine learning principles introduced by early work in this field (Jain et al., 2000). More recently, researchers used a commercial IMU suit and a neural network for each robot joint to enable a human to teleoperate the full body of a Nao humanoid robot (Stanton et al., 2012). These related pieces of research all demonstrate that machine learning from IMU data can facilitate reliable near-real-time interpretation of human movement without the occlusion and lighting problems that often affect visual data.

Past work on playful spHRI also shaped our approach. Investigations of robot play activities like hugging (Kanda et al., 2004) and performing magic (Nuñez et al., 2014) inform our interaction design and analysis strategies. A study of the physical play activities people exhibit with a small humanoid robot further parallels our work and similarly performs activity recognition using IMU data (Cooney et al., 2010). Previous work on dancing robots additionally blends touch with social interaction, allowing a human dance partner to guide a robotic dancer (Kosuge et al., 2003). This play research influenced how we processed data, designed motion, and selected scenarios to investigate.

## Hand motion classification

We previously demonstrated that a machine-learning pipeline trained on data from hand-worn IMUs can reliably classify hand-clapping motions (Fitter and Kuchenbecker, 2016c). In this past work, the two IMUs were attached to the backs of the human participant's hands using skin-safe adhesive. This attachment method did not always succeed in the presence of hair or sweat, it did not let the participant comfortably contact the robot with the backs of their hands, and it did not allow for easy removal of the sensors during breaks in the experiment.

Before building on our hand motion classification work, we needed a more robust and convenient way to attach the IMUs to participants' hands. Once developed, the new attachment method needed to be validated to confirm that the new form factor enabled accurate hand motion classification. This section describes how we achieved these two tasks and compares this updated approach to our previous work.

### Motion classification methods

**Figure 1:**
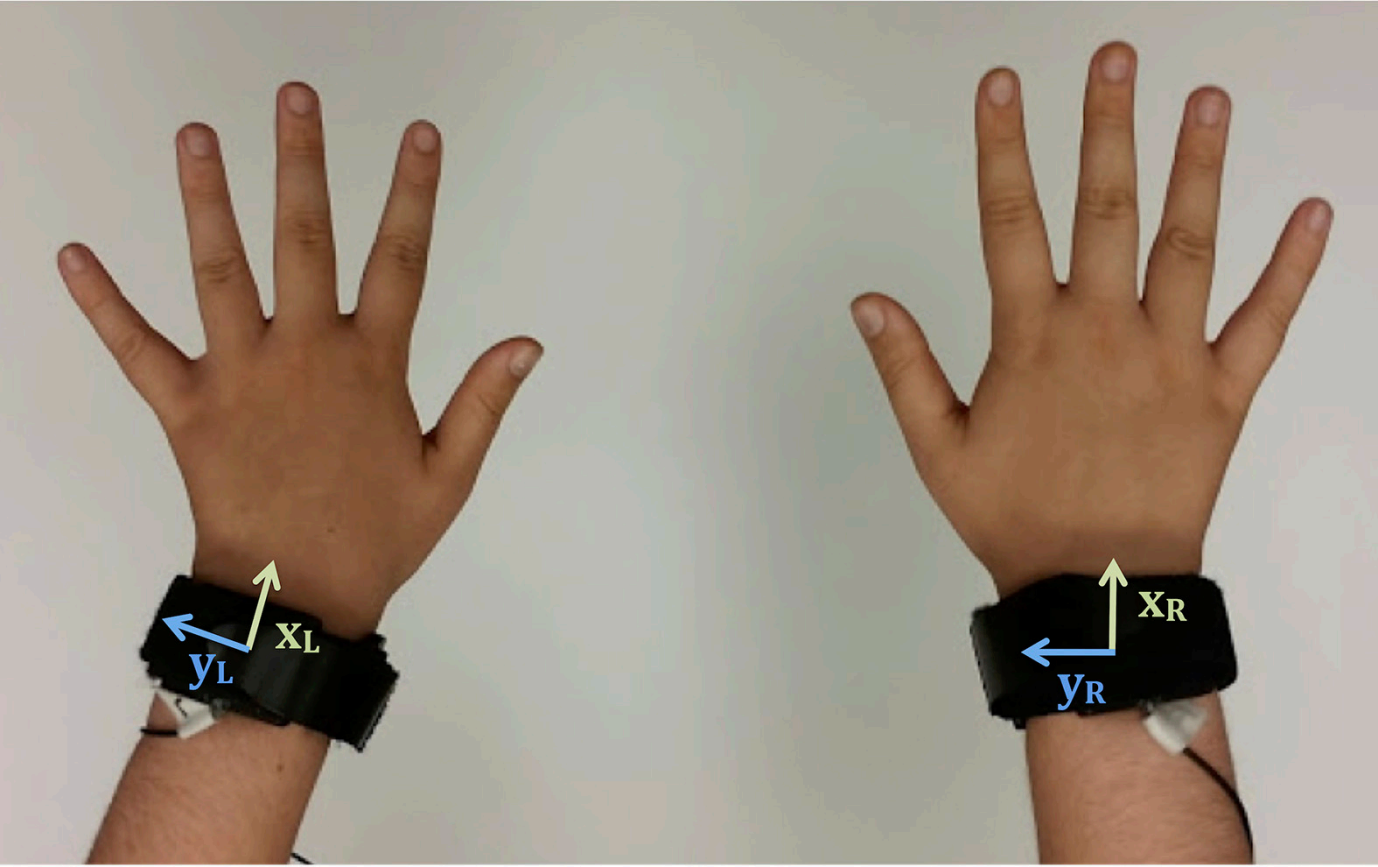
A plastic housing and integrated strap securely attach each inertial measurement unit to the user's wrist. The individual whose hands are shown in this image provided written consent for this image to be published.

In anticipation of intensive human-robot interactive gameplay scenarios, we chose to record participant motion via the same nine-axis Sparkfun MPU9150 IMU breakout boards used in prior work (Fitter and Kuchenbecker, 2016c). These sensors were affixed to each participant's wrists with Velcro straps that looped through custom 3D-printed housings, as shown in Figure 1. In addition to increasing the consistency and comfort of the sensor attachment, this scheme facilitated detaching and reattaching the sensors as needed during the experiment. While our sensors communicate via a lightweight cable, future iterations of this sensor system could be designed to use wireless communication.

With the sensors in this configuration, we aimed to classify hand motions using an updated version of the best method from our past work; it used *training and testing data* to create a linear support vector machine (SVM) that classifies individual hand-clapping motions based on particular features of the recorded data (Fitter and Kuchenbecker, 2016c). We slightly modified the set of target motions being learned to increase the diversity of hand-clapping games that could be constructed from them. This new set of motions requires wrist and hand movements that are largely similar to those studied in our prior work. However, relocating the IMU from the hand to the wrist prevents the system from observing the motion of the wrist joints and therefore reduces the expressivity of the captured data; thus it was possible that the wrist-worn sensors would necessitate a different type of data analysis.

#### Hand-clapping game selection

**Figure 2:**
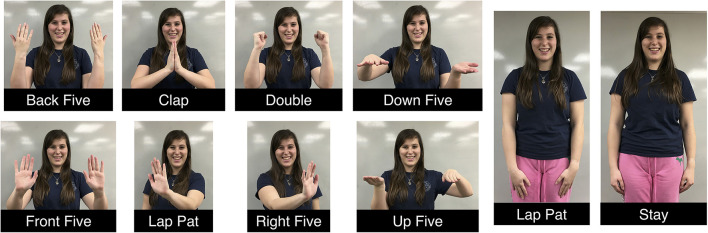
Labeled images of a person performing the studied set of hand-clapping motions. The individual shown in these images provided written consent for these images to be published.

This investigation of motion classification accuracy from wrist-worn IMUs involved 10 motion primitives. Nine of the motions were the same as primitives studied in our previous work (Fitter and Kuchenbecker, 2016c), and one motion was new. Our previous investigations discovered that many participants were not able to snap their fingers, and also that people tended to pause at specific parts of various hand-clapping games. Accordingly, our updated experiment traded the previously used “right snap” motion for a stationary “stay” motion. Figure 2 shows the set of primitives used in this investigation: back five (B), clap (C), double (D), down five (DF), front five (F), lap pat (LP), left five (L), right five (R), stay (S), and up five (UF).

To investigate the overall performance of prospective classifiers, we needed to select several hand-clapping games that use sequences of our chosen motion primitives and offer a range of classification challenge levels. This data collection considered the following six hand-clapping games, half of which are different from the patterns used in our previous work:

- Pat-a-cake: LP-C-R-C-L-C
- Slide junior: C-R-C-L-C-B-F
- Double double: D-D-F-F-D-D-B-B-D-F-D-B-D-D-F-B
- Down up clap: DF-UF-C
- Sailor: C-R-C-L-C-F-F-F
- We will rock you: LP-LP-C-S

In each of these hand-clapping games, pairs of people typically repeat the listed motions over and over along with a verbal chant. For the purposes of this investigation, a single person outfitted with sensors instead pantomimed the motions alone, in the style of someone who is teaching their partner a new hand-clapping game. This approach allowed us to first focus on classifying motions and later add layers of complexity to the interaction.

#### Human hand-clapping behavior

We conducted an experiment to collect a rich dataset for automatic classification of hand-clapping motions. Ten participants enrolled in our data collection, gave informed consent, and successfully completed the experiment. The University of Pennsylvania Institutional Review Board (IRB) approved all experimental procedures under protocol 822527. No formal demographic survey was administered in this data collection, but experimenter notes show that the participant population was composed entirely of technically trained students who all possessed normal motor function in their arms and hands. Each participant came to the lab for a single session that lasted about 30 minutes. The participant's wrists were outfitted with IMUs as shown in Figure 1. The raw x, y, and z-axis accelerometer, gyroscope, and magnetometer readings from both wrists were read by an Arduino Teensy and sent to our data processing program via a USB connection at 200 Hz.

We recorded two datasets from each participant: (1) a *training set* that contained selected pairs of motions repeated 10 or more times and (2) a *test set* with each of the six hand-clapping games repeated three or more times in sequence. Training data were used for model training and cross validation, while testing data were reserved for a separate round of model evaluation. The training set was designed to include all 17 pairs of sequential motions that appear in the chosen hand-clapping games. Some of these pairs consist of the same motion repeated over and over, while the rest show transitions between two different hand-clapping motions.

### Motion classification results

We sought to discover whether our system could classify all of the recorded hand-clapping motions using sensor data recorded from the wrist-worn IMUs. In order to classify each hand-clapping motion, we parsed full IMU recordings into individual hand-clapping motion data segments by applying a first-order Butterworth high-pass filter with a cutoff frequency of 25 Hz to the root-mean square (RMS) of the x- and z-axis accelerations from both IMUs together. Local maxima finding on the resulting signal proved effective for identifying the center of each hand clapping motion, assuming consistent participant clapping tempo and correct execution of hand-clapping motions.

We applied the linear SVM technique that was found to most accurately classify hand motions in our previous work (Fitter and Kuchenbecker, 2016c). From each motion recording, we extracted a feature set composed of basic statistical measures (maximum, minimum, mean, variance, skewness, and kurtosis) from each x-, y-, and z-axis channel of the accelerometer and gyroscope, the RMS acceleration for each hand, and high- and low-pass filtered data from each of these channels (cutoff frequency of 25 Hz). As in prior work, we did not use the magnetometer because its readings were found to be unreliable in the indoor setting of the data collection. We also added a new set of Boolean features that indicate whether the measured acceleration range along each axis was greater than a threshold of 0.8 g. This new set of features was designed to detect changes in hand orientation that could help distinguish a clap from a lap pat after systematic errors distinguishing between these two motions in our previous work. A leave-one-subject-out cross-validation (LOSOCV) technique during model training let us compute a generalizable training-set classification accuracy. We also computed the test-set classification accuracy using the trained models. All calculations were performed in Python with the scikit-learn library using the default settings.

**Figure 3:**
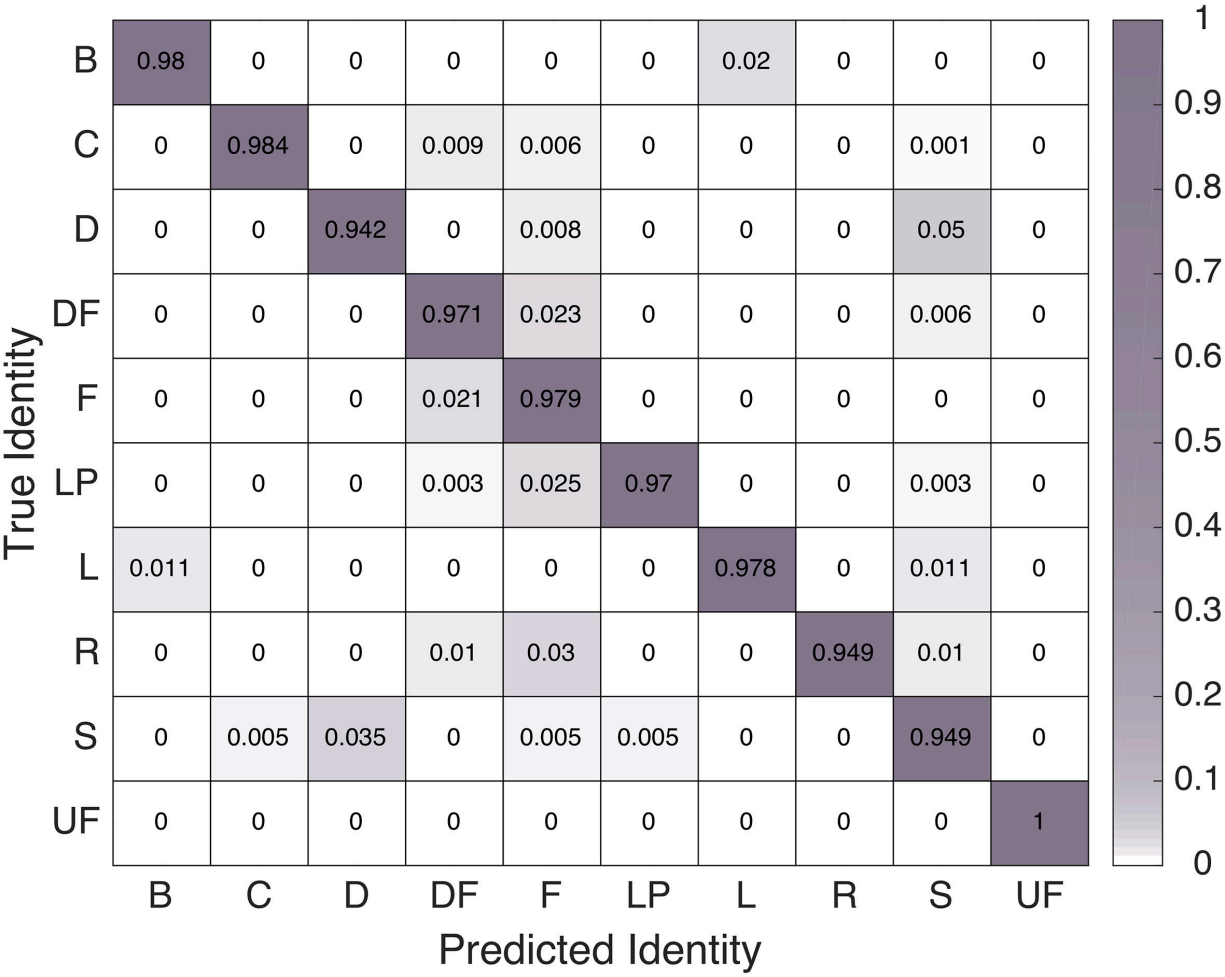
Confusion matrix of linear SVM classifier performance on the training dataset.

**Figure 4:**
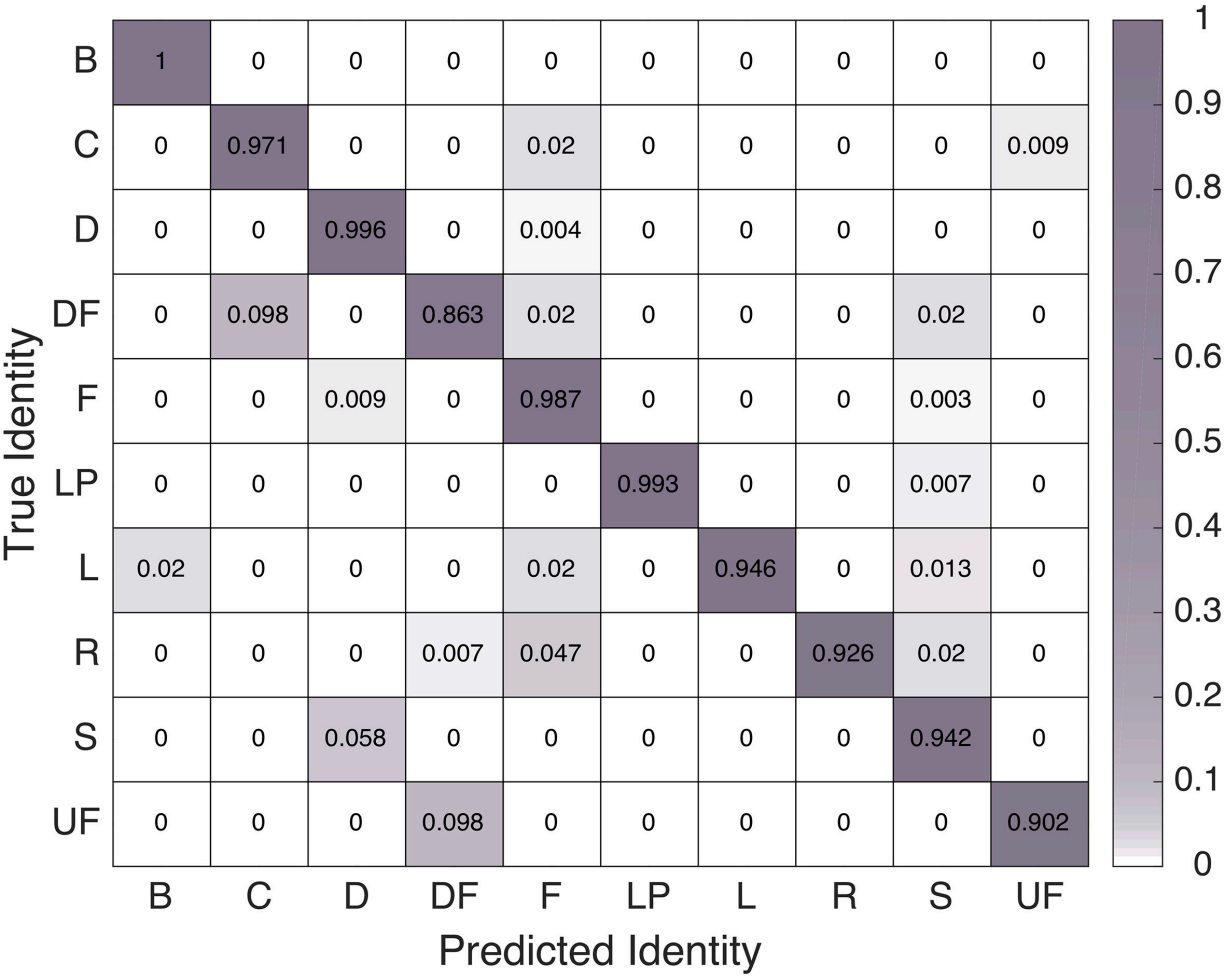
Confusion matrix of SVM classifier performance on the test dataset.

We examined the confusion matrices for this model's performance on the parsed training feature set and the parsed test feature set, as seen in Figures 3, 4, respectively. The 97.3% overall training-set accuracy stems from high values along the diagonal of the training confusion matrix, indicating excellent performance. Similarly, the 97.0% overall test-set classification accuracy stems from the strong diagonal of the test confusion matrix. Note that the 10 motions are not exactly evenly represented in either the training or testing set, so the two overall accuracy values differ slightly from the averages of the diagonal entries in the two confusion matrices

The overall classification accuracies indicate that the linear SVM classification strategy that worked best in our previous work also performs very well on data gathered from wrist-worn IMUs. The negligible difference between training and testing accuracies further shows that this technique generalizes well to hand-clapping motions performed as part of a longer sequence. Thus, this is the classifier we employed to enable our robot to understand motions pantomimed by a human partner.

## Hand-clapping study methods

We conducted a study to explore how people perceive different leadership and game generation experiences during bimanual hand-clapping interactions with a robot. The University of Pennsylvania IRB approved all experimental procedures under protocol 825490. Motivated by the desire to understand how our IMU machine learning pipeline can fit into meaningful spHRI applications, we were especially curious to discover what roles people prefer to play in these types of interactions, how structured or open-ended the interactions should be, and how users respond to inevitably varied machine learning performance.

### Hardware systems

**Figure 5:**
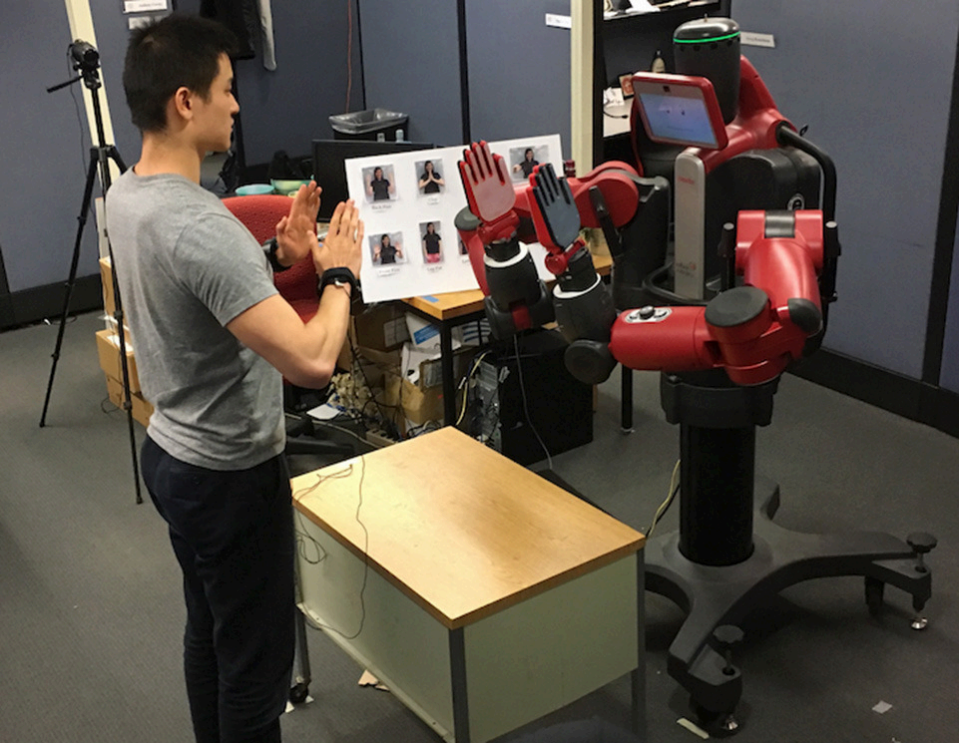
The experiment setup for the bimanual human-robot hand-clapping study. The individual shown in this image provided written consent for this image to be published.

This study centered on two MPU9150 9-DOF IMU sensors strapped to the wrists of a human user. The same 12 channels of IMU data discussed previously (x, y, and z-axis accelerometer and gyroscope readings from each hand) were transmitted from an Arduino Teensy to our data processing program via a USB connection at 200 Hz. The robotic agent for this investigation was a Rethink Robotics Baxter Research Robot, a sturdy human-sized platform that can exert human-level forces on the user's hands and can bear hand contacts without breaking or falling over. Our Baxter robot was equipped with two non-articulated custom hands, as shown in Figure 5. These custom hands are 3D-printed and covered with flexible silicone rubber, as presented in our previous work (Fitter and Kuchenbecker, 2016b). A small rolling table was placed between Baxter and the participant to both provide a lap-like surface against which Baxter could tap for the lap pat (LP) motion and to keep the user at a constant distance away from the robot.

To equip Baxter with knowledge of how to perform each hand-clapping motion in the bimanual clapping games, we physically moved Baxter's arms to preparatory poses and action poses for each motion, aiming to imitate the poses of a person's arms during these actions. Our control strategy used the Baxter software development kit's raw position controller and trajectory planning using cubic interpolation between successive key poses to allow Baxter to move smoothly and fairly quickly while playing games with a person.

### Experiment setup

24 participants (14 male and 10 female) enrolled in our study and gave informed consent. Participants were aged from 18 to 38 years (*M* = 24.4 years, *SD* = 5.2 years) and were mostly technical students (18 technically trained students, 2 non-technical students, 2 technically trained research assistants, 1 technically trained engineer, and 1 non-technical homemaker). Sixteen of the robot users originated from the United States, three from China, two from India, two from South Korea, and one from Belgium. All participants had full function in their arms and hands. Twenty-two participants were right-handed, and two were left-handed. We did not exclude left-handed participants because the experiment activities have balanced right and left hand roles, and also because some left-handed users were included in the dataset used to create the classifier. To help situate our results, we requested information about each user's applicable experience using robots. Participant experience with robotics ranged from 0 to 94 (*M* = 65.25, *SD* = 23.11) out of 100, with 100 being highest, and the group's experience with Baxter spanned the full range from 0 to 100 (*M* = 35.79, *SD* = 30.97).

Each participant came to the lab for a single 60-minutes session. The user stood facing Baxter throughout the experiment (as illustrated in Figure 5) and played various bimanual hand-clapping games with the robot, making hand-to-hand contact with Baxter throughout, as two people would when playing hand-clapping games. At the beginning of the session, the experimenter read a script to relay relevant background information on Baxter, described the experiment interaction, and asked the user to complete an opening survey about their perceptions of Baxter. Next, the participant was led through two sample interactions, one in which Baxter taught the user a simple game (C-R-C-L), and one in which the user taught the same game to Baxter.

In the main experiment, the user played hand-clapping games with Baxter in four blocks that each contained three interaction trials of a particular game. Over these three repetitions, either Baxter or the user would repeatedly teach the same motion sequence in order to give their partner a chance to practice it and improve. The block conditions varied in leadership assignment and game spontaneity, but every taught or learned game was eight motions long. After each block, the user completed a survey about their perception of the interactions within that set of three repetitions. After the four blocks, the user entered a free-play mode during which they could teach Baxter additional games and/or learn more games from Baxter. Finally, the participant completed a closing survey followed by a brief demographic survey.

### Data processing pipeline

The machine learning pipeline for human-led trials waited for the user to demonstrate an entire hand-clapping game and then parsed and classified each demonstrated hand-clapping motion from the full game recording. To help the pipeline identify meaningful portions of IMU data, we divided the experiment into discrete gameplay interactions that were fairly structured. At the beginning of a human-led trial, the experimenter asked the human user to be very still. When ready, the user would demonstrate the hand-clapping game to Baxter at the tempo of an ambient metronome that was set to 75 BPM. We relied on the participant pantomiming game motions at close to the metronome's tempo to give the motion parser a good guess of the inter-motion time interval. After the demonstration was complete, the user would return to being still and the experimenter would press a key on the Baxter workstation to relay the information that the demonstration was over.

At this point, the processing algorithm would have all the data from the human hand-clapping game demonstration. Thresholding on the gyroscope signal helped to determine precisely when the game demonstration started and stopped, which we took to be the transitions from stillness to general hand motion and general hand motion back to stillness. Within the portion of data identified to be the hand-clapping game demonstration, we could again use the first-order Butterworth high-pass filtered RMS acceleration of the x- and z-axis accelerations from both IMUs together to parse the motion recordings. Finding the local maxima of the resulting signal, combined with the knowledge of the stimulus spacing from the ambient metronome tempo, had seemed to be a good tool for identifying the center of each hand clapping motion when we tested this experiment with pilot participants. As in section Hand Motion Classification, the midpoints between local maxima were assumed to be the motion starting points.

Once the motion data was parsed, each section of data believed to represent a single hand-clapping motion was ready to undergo the feature extraction and classification processes outlined in section Hand Motion Classification. After the extraction of the features mentioned previously, the hand-clapping motion was classified using the linear SVM model trained in section Hand Motion Classification. Classified sequences of motions were reciprocated by the Baxter robot after the data processing step, for the final result of clapping gameplay with the user.

### Conditions

To begin understanding natural-feeling human-robot hand-clapping gameplay interactions, we needed to create opportunities for both Baxter and the user to lead complex interactions. We also aimed to strike a balance between well-controlled data collection and spontaneous natural play. Accordingly, we designed the experiment interactions to vary leadership assignment and spontaneity across trials. All other aspects of Baxter's behavior were kept as consistent as possible from trial to trial.

#### Leadership conditions

**Figure 6:**
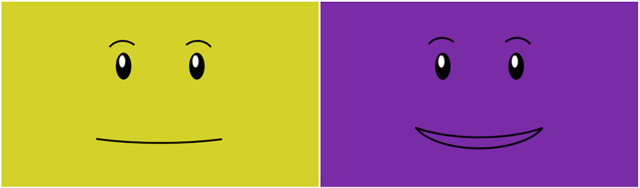
The two Baxter facial expressions used in this bimanual clapping study.

In each block of hand-clapping game interactions, either Baxter or the human user was assigned to lead the game. When Baxter was the leader, it demonstrated eight hand-clapping motions while displaying a yellow neutral face, and then it smiled, changed to displaying a purple face, and repeated the same eight motions, this time making physical contact with the hands of the user. Within a block, this process was repeated three times with the same hand-clapping game to promote human mastery of that particular game. The facial expressions used in the study were adapted from the Baxter Open-Source Face Database (Fitter and Kuchenbecker, 2016a) and appear in Figure 6.

When the participant was leading, they demonstrated a sequence of eight hand-clapping game motions to a metronome beat, paused briefly while Baxter “thought” about the motions, and then played the game with Baxter, making physical contact with the robot. Again, within a block, this process was repeated three times with the same hand-clapping game to promote robot mastery of that particular game. Baxter again showed the yellow neutral face during the demonstration and the purple happy face when it was time for interactive play.

#### Spontaneity conditions

When people play hand-clapping games with one another, the interaction often begins with the swapping of known hand-clapping game activities and then gradually becomes more complex or inventive. To promote this same type of natural development over the course of this experiment, we introduced a second “spontaneity” condition variable.

In the non-spontaneous interactions, the game leader (Baxter or the human participant) was instructed to teach a specific game to the other party. For Baxter, this instruction was delivered in code, and for the human user, it was delivered via verbal instructions from the experimenter. Two specific games were used for the non-spontaneous interactions: (1) Game A: LP-C-R-C-L-C-B-F and (2) Game B: D-F-D-B-D-D-DF-UF. If Baxter taught the user Game A, the user would teach Baxter Game B, and vice versa. The games were randomly assigned and balanced across users to prevent a confound between the conditions and the game motion sequence itself.

When the person was leading non-spontaneous gameplay, Baxter did not use the data processing pipeline to attempt to identify and reciprocate the human motion pattern. Instead, Baxter performed pre-set routines with two canned mistakes in the first repetition, one canned mistake in the second repetition, and none in the final repetition. The mistakes were consistent for each non-spontaneous game and were designed based on common machine learning classifier errors. This behavior ensured that even if our IMU system did not work well in this new application, we would be able to understand how a consistently improving robot would be received by human users. Additionally, the human wrist IMU data was recorded during these trials, which allowed us to include the would-be accuracy of the data processing pipeline's classification of these patterns in our overall machine learning results.

During spontaneous gameplay, games were still required to be eight motions long and had to begin with either a clap or lap pat as those two bimanual movements provide a distinct beginning signal in the recorded data. Otherwise, Baxter and the participant were free to choose their own sequence of hand-clapping motions from the set given in section Hand Motion Classification, minus “stay,” which was omitted because pilot participants had difficulty maintaining the rhythm when the sequence included this pausing move. To generate a random new game, Baxter employed a random number generator and a transition matrix of typical hand-clapping game motion transitions to create its own pattern. In human spontaneous lead cases, the user was free to create a game that followed the few guidelines mentioned above. Across the three interactions in a spontaneous play block, the robot and person were expected to repeat the same game to foster mastery by the team.

#### Overall block flow

To maintain an organic interplay throughout the experiment and allow the user to master the robotic system in the limited time available, we used the same block order for all participants. We present both the disadvantages and the advantages of this experiment structure in the discussion of this article. The order of the interaction blocks was always as follows:

1. Baxter-led non-spontaneous
2. Human-led non-spontaneous
3. Baxter-led spontaneous
4. Human-led spontaneous
5. Free play

This order gradually increased the autonomy of each partner while giving the human user time to become familiar with the system before leading an interaction. The transfer of leadership back and forth mimicked the natural tendency of people to take turns teaching their own clapping games when exchanging oral cultural traditions.

#### Data collection

**Figure 7:**
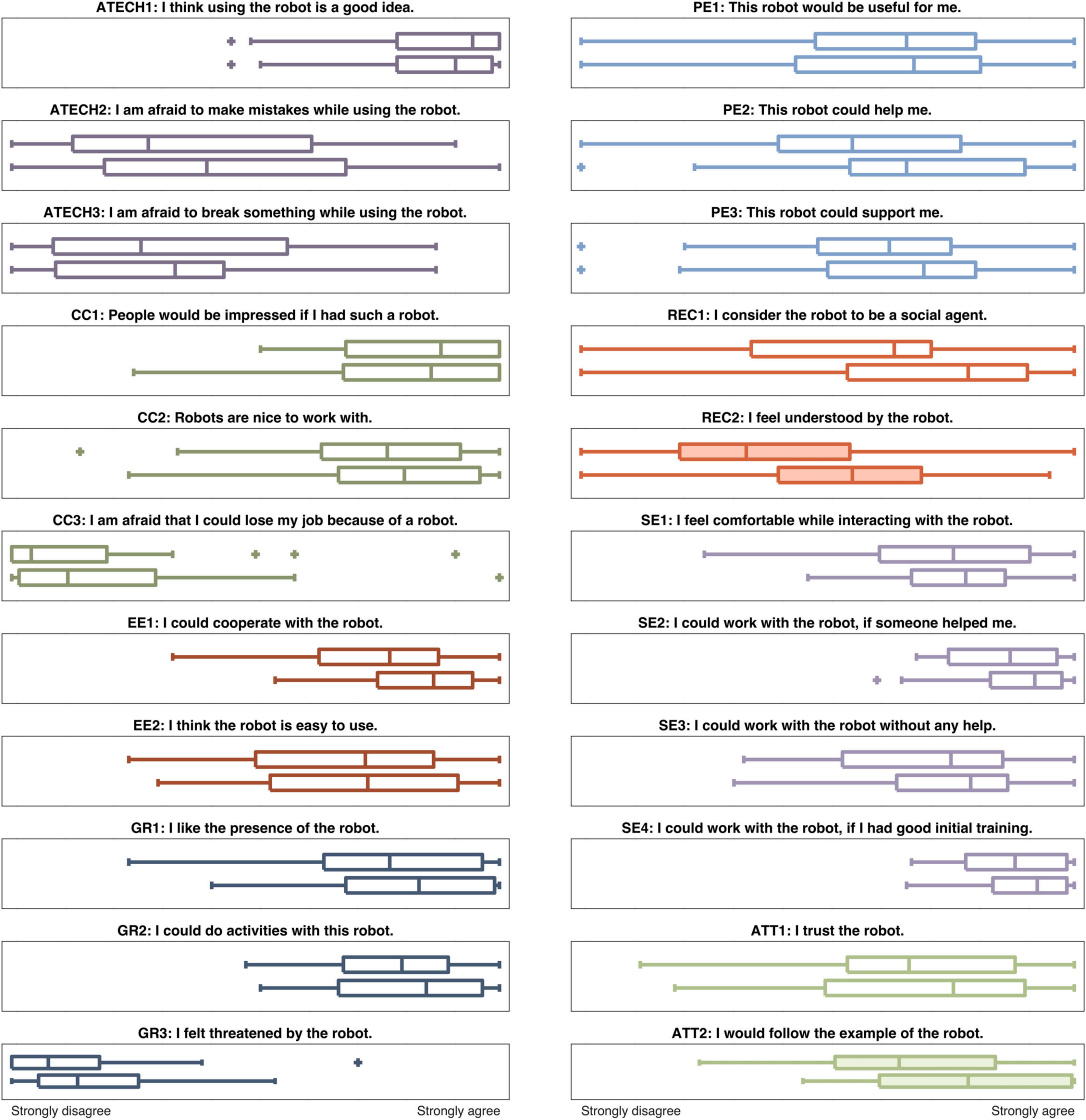
Differences in participant responses before and after the human-robot hand-clapping experiment. In each subplot, the upper box plot represents pre-experiment responses and the lower box plot represents post-experiment impressions. Filled-in box plots indicate significant differences. The question coding abbreviations stand for attitude toward technology (ATECH), cultural context (CC), effort expectancy (EE), forms of grouping (GR), performance expectancy (PE), reciprocity (REC), self-efficacy from UTAUT model (SE), and attachment (ATT).

Our software recorded the IMU data from the human user and the sequences of motions performed by Baxter. We also asked participants to complete four surveys: (1) a robot evaluation after hearing introductory information about Baxter, (2) an interaction block survey after each trio of hand-clapping game repetitions, (3) a concluding survey after the final free-play interaction, and (4) a basic demographic survey after the concluding survey. The block perception survey used questions from the pleasure-arousal-dominance (PAD) emotional state model (also used by Ammi et al., 2015), The National Aeronautics and Space Administration (NASA) task load index (TLX) (Hart and Staveland, 1988), and an enjoyability survey used by Heerink et al. (2008), plus a safety rating question, as displayed in Table 1. Later in this article, we bundle the PAD and safety questions together under the acronym “PADS.” Questionnaires (1) and (3) were adapted from the unified theory of acceptance and use of technology (UTAUT) and other metrics employed by Weiss et al. (2008) and Heerink et al. (2009); the questions are shown in the plot titles of Figure 7. The block survey and concluding survey also included the following free-response questions to help elicit experiential information from users:

- What aspects of this activity did you enjoy?
- What aspects of this activity were most challenging?
- Why would or wouldn't you want to do this activity with a robot?
- What other activities would you want to do with this robot?

**Table 1:** Content of the block questionnaire used in the bimanual clapping study.

***Block Evaluation***	
***Please rate the following on the provided sliding scales:***	
How safe did this robot behavior seem?	How engaged did you feel throughout this set of interactions?
How pleasing is this robot behavior?	How well did you perform during this set of interactions?
How energetic is this robot behavior?	How well did the robot perform during this set of interactions?
How dominant is this robot behavior?	How rushed did you feel during this set of interactions?
How much did you enjoy this set of interactions?	How calm did you feel during this set of interactions?
***Free response section. Please respond briefly to the following questions:***	
Describe your experience interacting with the robot in this way, including any positive or negative aspects of the experience.	

The experiment was additionally videotaped for later analysis of user and robot behavior.

#### Hypotheses

This experiment sought to test the four main hypotheses detailed below:

- **H1**: *Users will enjoy teaching hand-clapping games to Baxter as much as learning games from Baxter*. In human-human game interactions, some people prefer to lead and others prefer to follow the lead of others. Some individuals may enjoy both leading and following depending on the interaction scenario. Because of this balance of preferences, we believed that people might rank robot pleasantness and interaction enjoyment the same regardless of who leads the game.
- **H2**: *Participants will find spontaneous hand-clapping interactions more fun and engaging than scripted ones*. In this experiment's prototyping and piloting phases, we originally considered scripting all of the hand-clapping actions throughout the experiment blocks, but pilot users expressed a strong desire to create and teach their own hand-clapping games. This feedback led us to modify the experiment protocol into the currently described state. We additionally wanted to test this hypothesis to ascertain whether the pilot user preferences generalize to other users.
- **H3**: *Participation in the experiment will alter the way people perceive the robot*. Specifically, we administered a UTAUT-inspired survey before and after the experiment to determine whether the interactions caused any changes in user perception of Baxter. Playing games with Baxter in this study might be a pleasant or unpleasant social experience that would alter later user responses.
- **H4**: *Our proposed machine learning pipeline will perform well at classifying hand-clapping motions in this new use scenario with a robot in the loop*. Our machine learning strategies performed well on previous test datasets, but we wanted to test whether the linear SVM classifier would provide similar motion classification accuracy in a more realistic and demanding interaction scenario.

These hypotheses helped guide the design of the experiment blocks and the interactions described previously in this section.

## Results

All 24 users who enrolled in the study successfully completed the experiment. 23 of them were willing to physically contact the robot to play hand-clapping games. The other one person was bothered by the sound of Baxter's motors and only occasionally clapped hands with the robot; this individual's data were not excluded from the analysis because they still took part in the entire experiment.

This section focuses on statistical analyses of the questionnaire responses using paired *t*-tests and repeated measures analysis of variance (rANOVA). The *t*-tests enable us to discover whether the experiment changed user opinions of Baxter. The rANOVAs (using the R “aov” function and an α = 0.05 significance level) tell us how different hand-clapping game experiences affected block survey responses on the PADS, enjoyment, and TLX questionnaire scales. We also consider overall user comments and the success of the hand-clapping game motion classifier.

### Before/after survey results

We gathered matched sets of robot perception survey responses before and after the experiment. The overall user responses appear in Figure 7. Paired *t*-tests reveal that the answers to two questions significantly changed. Namely, after the experiment participants reported feeling more understood by the robot (REC2: *p* = 0.023, *M*_before_ = 35.54, *M*_after_ = 52.33) and also more willing to follow the example of the robot (ATT2: *p* = 0.031, *M*_before_ = 65.29, *M*_after_ = 78.04). Additionally, user ratings on the overall reciprocity-focused questions were higher after the experiment than before (REC1 + REC2: *p* = 0.010, *M*_before_ = 45.35, *M*_after_ = 60.52).

### Block survey results

The within-subjects factor for our rANOVA was game block condition, giving a design space of four blocks with a cross of two leadership conditions and two cooperation conditions. We had initially designed the block differences as a 2 by 2 space, but after running the experiment, we realized that ordering played a role in the users' perceptions and that experiences in the paired conditions were sometimes quite different. Accordingly, we concluded that the most appropriate analysis tool was a one-way rANOVA comparing the four different block conditions as distinct levels of the factor. When an effect was significant for a particular outcome measure, *post-hoc* multiple comparison tests using the R “multcomp” library revealed which pairs of conditions had statistically significant differences. We also calculated the effect size using eta squared.

The rANOVA results for the block survey are summarized in Table 2, and breakdowns of interaction block effects on different question groupings appear throughout the following paragraphs.

**Table 2:** *p*-values for the one-way rANOVA run to determine the effects of the block conditions.

	**Safety**	**Pleasantness**	**Energeticness**	**Dominance**	**Enjoyment**	**Engagement**
Block	0.093	0.022	0.500	0.004	0.061	0.146
		**Human Perf**.	**Robot Perf**.	**Lack of Rush**	**Calmness**	
	Block	0.104	<0.001	0.702	0.057	

*Gray shading indicates a statistically significant effect*.

#### PADS results

**Figure 8:**
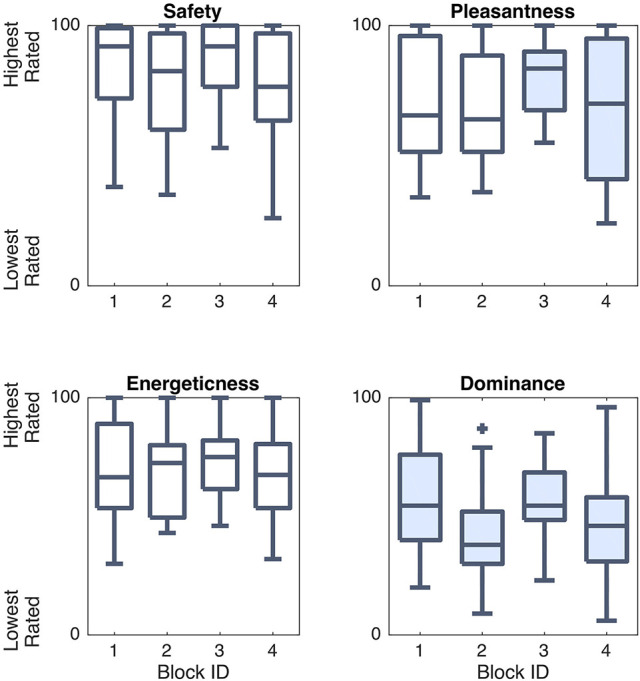
Differences in responses to the PADS survey questions across interaction blocks. Shaded boxes are significantly different from at least one other condition.

We were curious to know how each block condition affected user ratings of safety and affective characteristics of the robot behavior, so we performed a one-way rANOVA for each of the PADS survey questions. There were several statistically significant trends in these block survey question responses, as outlined in Table 2 and Figure 8.

Block modes significantly affected user ratings of robot pleasantness [*F*_(3, 69)_ = 3.88, *p* = 0.022, η^2^ = 0.058] and dominance [*F*_(3, 69)_ = 5.94, *p* = 0.004, η^2^ = 0.105]. *Post-hoc* multiple comparison tests revealed that Block 4 (human-led spontaneous) was rated as less pleasant then Block 3 (robot-led spontaneous). Block 2 (human-led non-spontaneous) made Baxter appear less dominant than Block 3, while Block 1 (robot-led non-spontaneous) made Baxter appear more dominant than Blocks 2 and 4. No significant differences were found for safety or energeticness, and safety ratings were uniformly high (*M* = 79.71, *SD* = 21.59).

#### Enjoyment results

**Figure 9:**
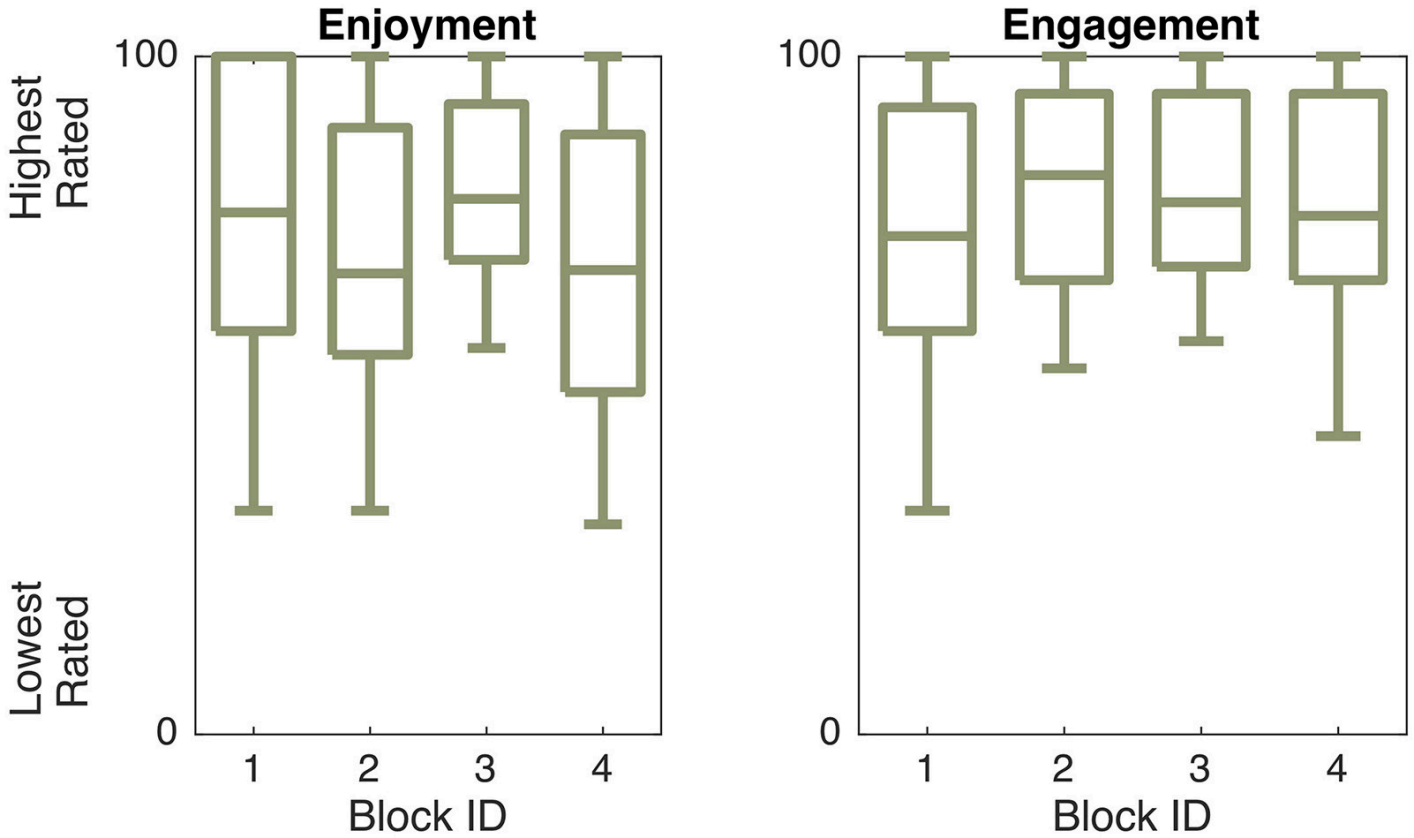
Responses to enjoyment-related survey questions across interaction blocks. No significant differences were found.

We also wanted to know how game block experiences influenced user ratings of enjoyment and engagement, so we performed a one-way rANOVA for each of the related block survey questions. There were no statistically significant trends in these responses, as shown in Table 2 and Figure 9. Enjoyment (*M* = 74.25, *SD* = 19.83) and engagement (*M* = 78.59, *SD* = 16.75) were both uniformly rather high.

#### TLX results

**Figure 10:**
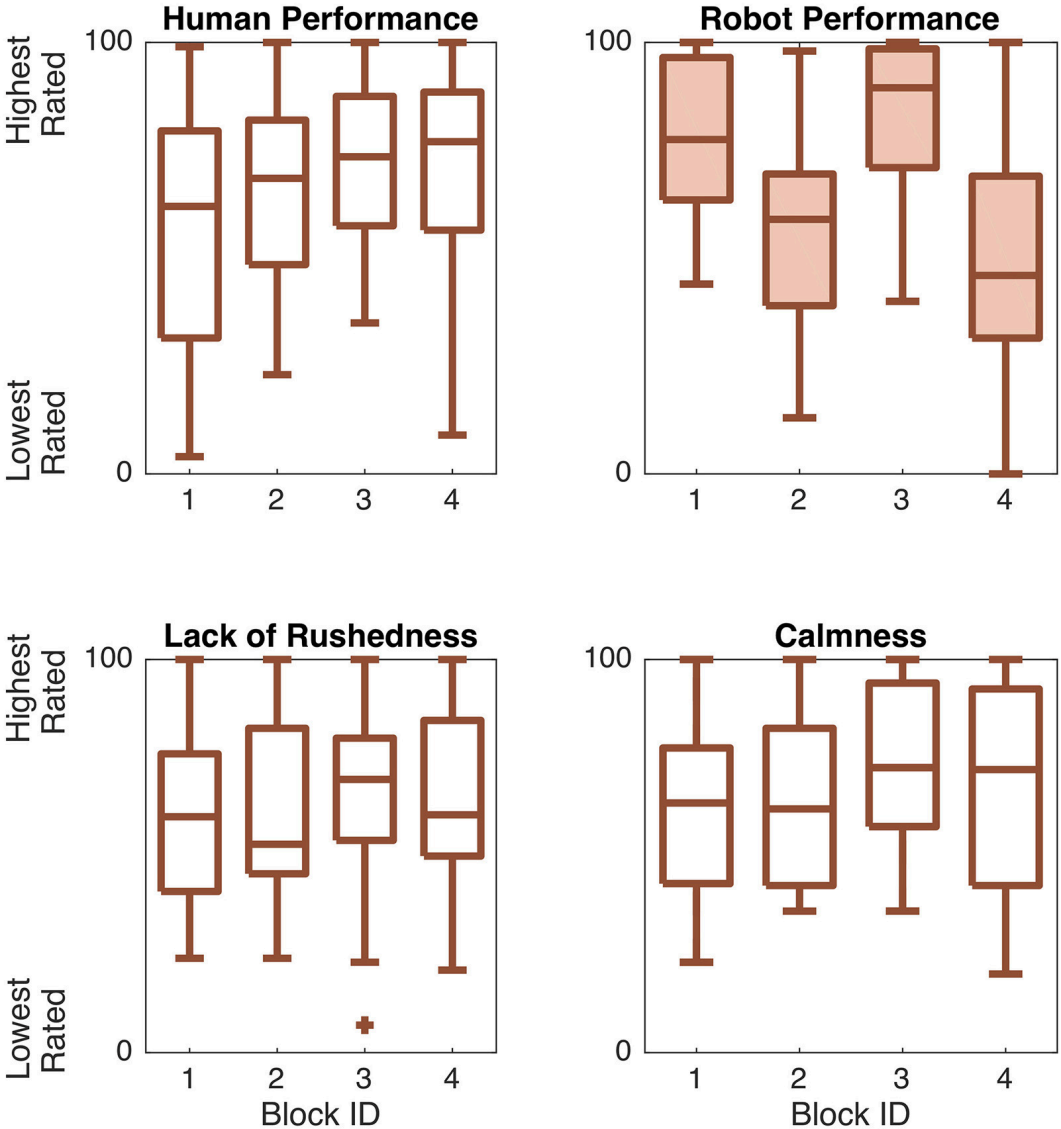
Differences in responses to TLX-related survey questions over interaction block. Shaded boxes are significantly different from at least one other condition.

Lastly, we looked to identify how game block experiences influenced user ratings of various task-load metrics. We performed a one-way rANOVA for each of the TLX-inspired block survey questions. There was one statistically significant trend in the responses, as depicted in Table 2 and Figure 10.

Block modes had statistically significant effects on user ratings of robot performance [*F*_(3, 69)_ = 18.95, *p* < 0.001, η^2^ = 0.332]. The difference in the ratings of block interaction calmness was also close to significant [*F*_(3, 69)_ = 2.90, *p* = 0.057, η^2^ = 0.045]. A *post-hoc* multiple comparison test revealed that robot performance appeared to be better in both robot-led blocks (Blocks 1 and 3, *M* = 80.65, *SD* = 17.04) than in both human-led blocks (Blocks 2 and 4, *M* = 52.71, *SD* = 23.72). No significant differences were found for human performance, rushedness, or calmness.

### Participant demographic results

Differences in participant feedback can stem from either study conditions or characteristics of the users themselves. To investigate differences due to participant demographics, we performed a further set of rANOVA tests with survey timing or block condition as a fixed factor and participant gender and region of origin as covariates.

Gender had a significant effect on several user ratings. Women thought people would be more impressed by their ownership of Baxter than men did [*F*_(1, 23)_ = 4.60, *p* = 0.038, η^2^ = 0.084]. Female participants also liked the presence of the robot more [*F*_(1, 23)_ = 7.69, *p* = 0.008, η^2^ = 0.146] and thought they could do activities with the robot more [*F*_(1, 23)_ = 7.13, *p* = 0.011, η^2^ = 0.134] than male users. Women were additionally more willing to follow the example of the robot [*F*_(1, 23)_ = 19.75, *p* < 0.001, η^2^ = 0.279]. Female robot users also found the robot more pleasant [*F*_(1, 23)_ = 10.14, *p* = 0.002, η^2^ = 0.095], found the interaction more enjoyable [*F*_(1, 23)_ = 11.00, *p* = 0.001, η^2^ = 0.104], felt more engaged during the study [*F*_(1, 23)_ = 8.75, *p* = 0.004, η^2^ = 0.085], and felt more rushed during the interactions [*F*_(1, 23)_ = 11.15, *p* = 0.001, η^2^ = 0.108].

Since Eastern and Western cultures tend to have different views of robots and other technologies (Lee et al., 2012), we were also interested in comparing participant responses across origin lines. Robot users from Eastern cultures thought others would be more impressed by their possession of Baxter than those from Western cultures [*F*_(1, 23)_ = 5.68, *p* = 0.021, η^2^ = 0.104]. Individuals from Eastern cultures also found the robot more dominant than Western participants [*F*_(1, 23)_ = 6.81, *p* = 0.011, η^2^ = 0.0626].

### User comments

**Figure 11:**
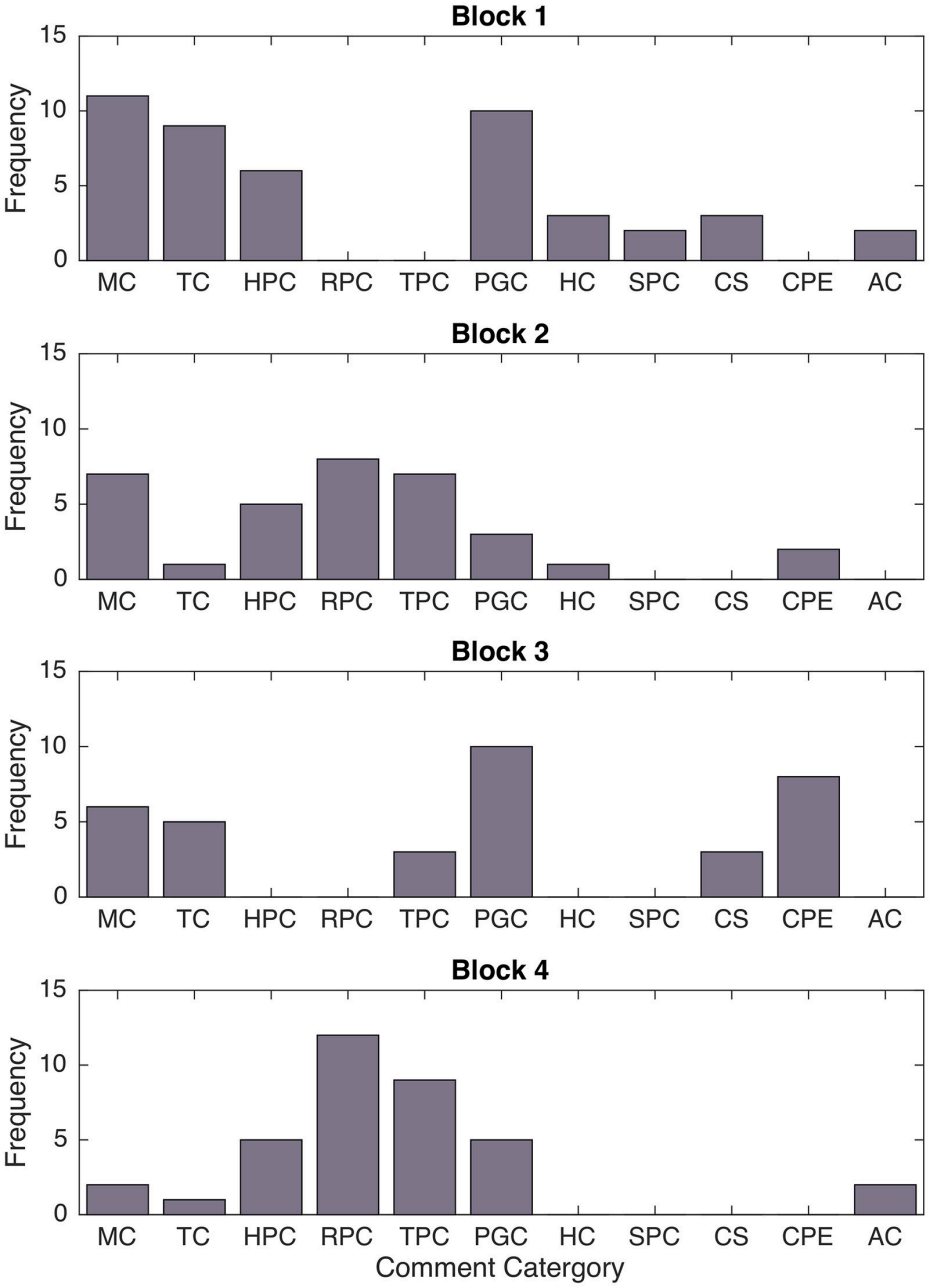
Comparison of frequency of comments pertaining to various topics during different interaction blocks. The topic codes were: motion comments (MC), temporal comments (TC), human performance comments (HPC), robot performance comments (RPC), teamwork performance comments (TPC), positive general comments (PGC), haptic commentary (HC), social performance comments (SPC), cue suggestions (CS), comparisons to previous experience (CPE), and additional clarifications about how users were reading survey questions (AC).

While analyzing user comments on each interaction block survey, we noticed the emergence of the following themes: motion comments (MC), temporal comments (TC), human performance comments (HPC), robot performance comments (RPC), teamwork performance comments (TPC), positive general comments (PGC), haptic commentary (HC), social performance comments (SPC), cue suggestions (CS), comparisons to previous experience (CPE), and additional clarifications about how users were reading survey questions (AC). Example comments from each topic code appear in Table 3. This division of comments seemed interesting, especially because the frequency of comments in each topic area shifted from block to block, as pictured in Figure 11. Some participants wrote multi-part comments that fit into several categories, as included in the frequency counts.

**Table 3:** Example comments from each interaction block topic code.

***Topic Code***	***Example Comment***
Motion Comments (MC)	“The ‘Up Five’ and ‘Down Five’ were a little low, making those interactions a little more awkward/unsafe feeling. That also probably has to do with the fact that the motions of the robot is much larger.”
Temporal Comments (TC)	“A small delay between Baxter's demonstration and actual task would have made it easier.”
Human Performance Comments (HPC)	“I was pretty bad at first (and at the end too) but improved with each trial.”
Robot Performance Comments (RPC)	“Robot made mistakes in the first round just like me! He finally learned it in the end, whew.”
Teamwork Performance Comments (TPC)	“The interaction was still fun, but I was confused as to whether or not I had shown Baxter the motions clearly enough or if Baxter just had trouble replicating that motion at that time.”
Positive General Comments (PGC)	“That one was fun!”
Haptic Commentary (HC)	“Claps felt a little soft, though not limp.”
Social Performance Comments (SPC)	“I am not sure how a robot could be energetic. I did not feel that it was dominant or pleasing.”
Cue Suggestions (CS)	“Playing a beat like the metronome from the teaching part would make staying on pace easier.”
Comparisons to Previous Experience (CPE)	“Baxter's motion seems more smooth and safe than last time.”
Additional Clarifications (AC)	“Any lack of calm is due to the metronome and my anxiety to remember the patterns.”

Overall, the human-led Block 2 and Block 4 experiences yielded more comments on the performance of the robot and the human-robot team than other parts of the experiment. Robot-led Blocks 1 and 3 led to an emphasis on motion and temporal commentary, as well as cue suggestions, perhaps because users were not as occupied with thinking about their own motions and demonstration success. Some comment frequency progressions may have occurred due to trial ordering effects; for example, the motion commentary may have decreased over the course of the experiment because users became accustomed to Baxter's movements. Other comments seem related to who was leading a trial, returning whenever a leadership condition occurs. The game spontaneity condition did not greatly affect user comments. Furthermore, the breakdown of comments in the canned “perfect robot improvement” performance of Block 2 is quite similar to that of Block 4, during which Baxter often still made mistakes in the final hand-clapping interaction.

### Free play results

In the free-play interaction following Block 4, all but two users identified a favorite interaction mode that they wanted to play again. The participants who chose not to engage in additional free play were not afraid of the robot; they simply were not interested in additional interactions at that time. One of them was the user who refrained from contacting the robot during the main blocks due to the robot's sound, and the other stated that they were more pedagogically curious about the robot than interested in the social aspects of play with it. All other participants played at least one more game repetition with Baxter during the free-play segment (2.2 game repetitions on average, with a range of 0 to 5 repetitions across the participant pool).

Participants varied in the types of additional interactions they wanted to perform with Baxter. Seven users both learned from and taught Baxter during the free-play time. Eleven users chose to only teach Baxter, while four opted to only learn from Baxter.

### Classifier results

Another goal of this bimanual hand-clapping study was to evaluate the performance of the motion classifier described in section Hand Motion Classification. Data recording errors occurred during the first four sessions of this experiment, so our classifier evaluation omits these participants. In the data recordings of the remaining 20 users, the following pre-processing steps were applied before evaluating the accuracy of Baxter's real-time motion labeling in the bimanual gameplay:

- Our strategy to detect the overall relevant segment of IMU data in human-led trials required participants to be very still before and after their motion pattern demonstration. Some participants were unable to be still, which resulted in extra motion classifications at the beginning or end of their demonstration, due to fidgeting or preparatory motions before the intended demonstration. These extraneous motions were identified via video review and omitted while evaluating classifier accuracy.
- Although human users were not allowed to choose the “stay” motion when teaching Baxter games, Baxter was permitted to classify human motions with this label. In piloting, we found that this class label helped Baxter adapt to minor human pauses or rhythmic inconsistencies; Baxter could pause during these incidents rather than performing the next most likely (incorrect) motion. Before comparing the actual and classified participant motion identities, we removed all of the “stay” padding occurrences.
- Another algorithmically problematic behavior occasionally performed by users was motion demonstrations at half of the suggested demonstration speed. This type of demonstration usually produced some intermittent “stay” classifications (as mentioned above) and some double- or triple-registers of individual motions. Any duplicate registers of motions caused by half-time hand-clapping demonstrations were identified by video review and removed from the classification labels before computing classifier accuracy.
- Lastly, the human experimenter controlled when the data recording for each demonstration stopped. She sometimes stopped recording data too soon, clipping the last hand motion recording and causing one motion label to be missing from the resulting motion sequence. In these cases, we evaluated only the prediction accuracy for the first seven demonstrated motions.

Generally, we were monitoring for the correct sequence of motions in the recordings, regardless of what occurred between consecutive moves.

**Figure 12:**
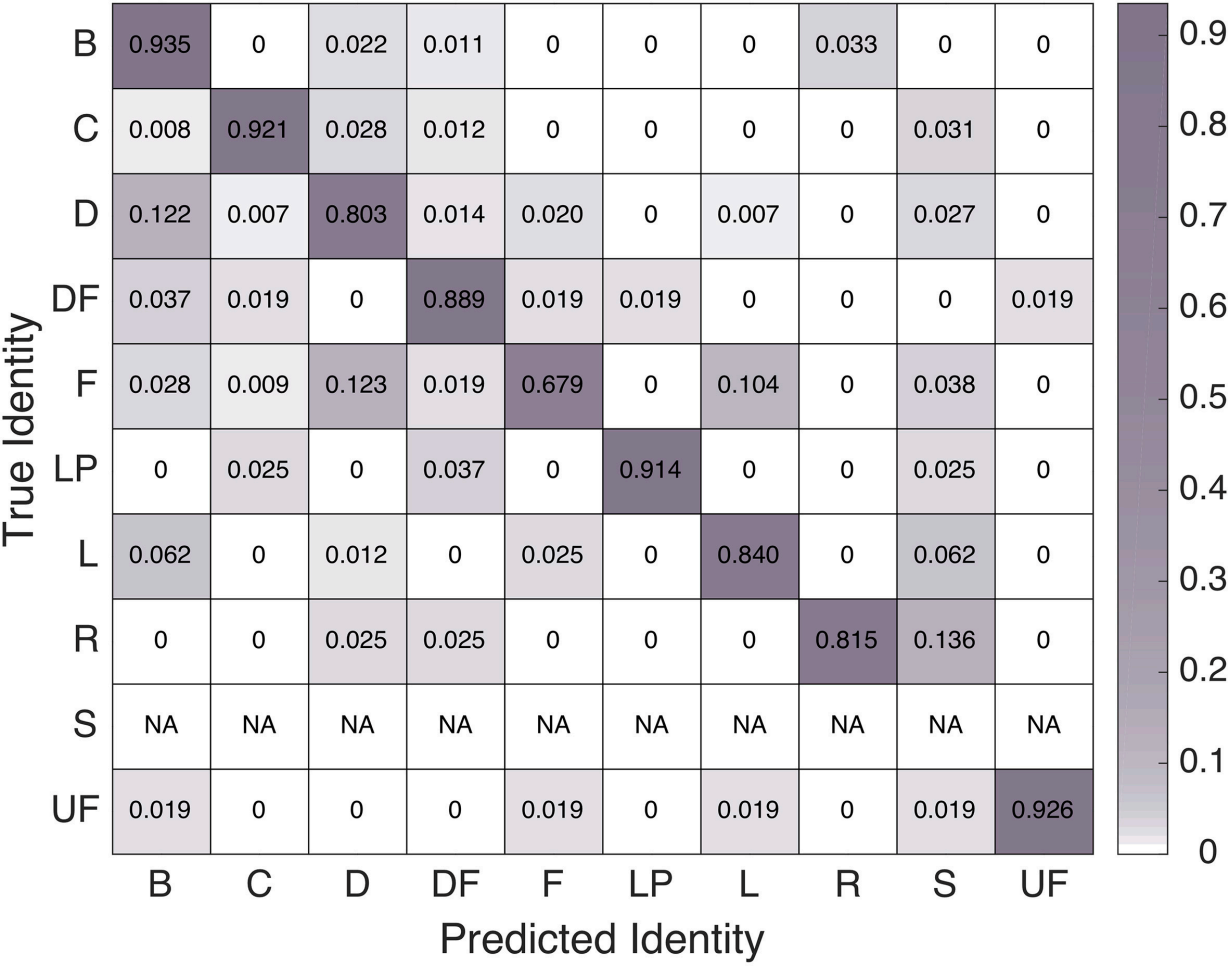
Confusion matrix of the linear SVM classifier's performance in the real experiment setting, after the four mentioned pre-processing steps.

After these data processing steps, we were able to compare the data processing pipeline's linear SVM classifications with the actual identity of each hand-clapping motion demonstrated by the human user (taken from the specified game sequence or the demonstrated sequence visible in the video). The overall accuracy of this classification was 85.9%, and the breakdown of correct and incorrect motion labels appears in Figure 12. Although high, this accuracy is to be taken with the caveat that even when our analysis interpreted 100% classification accuracy for a particular game, the user may have seen extra moves before or after their intended game, extra “stay” motions, duplicate motions, or missing final motions in Baxter's reciprocal motion pattern. Participants reacted to these behaviors and classification errors in a variety of ways, from adjusting their behavior to match Baxter's errors to questioning Baxter's sobriety. Errors that caused Baxter to perform worse in the consecutive game repetitions making up one study block were most frustrating to users.

## Discussion

The experimental results enable us to test our hypotheses and plan how to move forward with this spHRI research.

### Hypothesis testing

The **H1** prediction that users would enjoy teaching games to Baxter as much as learning games from Baxter was partially supported. There was no statistically significant difference in user ratings of Block 1 vs. Block 2 interactions on the robot pleasantness scale, but participants rated robot behavior in Block 3 (robot lead, game spontaneous) as more pleasant than Block 4 (human lead, game spontaneous). Despite this pleasantness difference, users most frequently chose to continue teaching the robot during the free-play time, rather than continuing to learn from Baxter. Interaction enjoyment ratings, on the other hand, did not differ significantly across any of these conditions. This finding might indicate that teaching to and learning from a robot that improves consistently (Blocks 1 and 2) are equally fun and pleasant activities, while a robot that displays different types of learning patterns is interesting but less pleasant. Another intuitive difference in robot dominance ratings appeared in the robot lead vs. human lead trial comparison; participants rated Baxter as less dominant when the robot was following their game lead, except in the comparison of Blocks 3 and 4, which did not yield a significant difference. Robot performance also received higher ratings for robot-led trials compared to human-led trials.

There was less evidence to support **H2**'s predicted preference for spontaneous hand-clapping activities. Overall, no block survey response difference emerged from the comparison of scripted and unscripted game experiences. When Baxter taught games to the user, the person never knew whether Baxter's motion sequence was pre-set, so it makes sense that the human perception of these game activities was fairly uniform. We thought that users might enjoy creating their own clapping game in the fourth experiment block, but experimenter notes show that some people were eager to undertake this task while others were quite intimidated by having to compose their own pattern. Participants who liked being able to teach Baxter commented that “it was fun to watch the robot trying to move in the way [they] created and taught,” “making up [their] own motion and seeing [Baxter] learn it made the experience more exciting,” and “it was more fun leading than learning from the robot.” Less enthusiastic users noted that they “had trouble teaching Baxter,” felt “anxiety from […] memorizing the pattern of clapping,” and wondered “whether or not [they] had shown Baxter the moves clearly enough.” These two viewpoints may have contributed to the lack of overall differences between Block 2 and Block 4 ratings.

Our hypothesis **H3** was correct. Users rated their perception of Baxter differently on the pre- and post-experiment surveys. Participant felt more understood by the robot after the experiment, and they also became more willing to follow Baxter's example. The overall feelings of reciprocity between participants and Baxter grew during the experiment as well, indicating that the robot successfully achieved at least a rudimentary form of social-physical interaction.

The final hypothesis **H4** predicted that our machine learning pipeline would perform well and help Baxter to understand human motion demonstrations throughout human-led interactions. We especially hoped that the classifier would work well in Block 4, during which Baxter had no information about the motion sequence that the human user would demonstrate. The classifier was able to label human hand-clapping moves with 85.9% accuracy. This recognition rate is lower than the 97.0% achieved on the testing set, and it has some additional caveats. Mainly, the data processing pipeline's motion parsing technique required users to demonstrate games at a specific constant tempo with no errors or hesitations. We acknowledge the need to improve classifier robustness and have additional new users test the system to confirm the redesign's success. Fortunately, the IMU data recorded throughout this study gives us a new prospective training set for improving our classifier's robustness to pauses and variable demonstration tempo in future bimanual clapping interactions. We hope to determine the maximum human motion recognition accuracy that can be achieved using IMUs in a natural setting.

### Major strengths and limitations

This study represents the most complex and natural-feeling HRI that we have investigated, and we were pleased with the promising and informative results. All participants successfully completed the study, and although one user never contacted Baxter through an entire cycle of hand-clapping motions, this individual's interaction displeasure arose from the timing of the noises Baxter produced, rather than concerns about the safety of the robot. This person wrote that “there seemed to be some feedback missing (for example, a sound to accompany the hands clapping), which damaged any sense [of] rhythm that might have driven the pace of the game.” Additionally, all but two of the users identified a free-play interaction that they wanted to try and engaged in that activity with Baxter during at least one additional round of hand-clapping gameplay.

This study interaction led to improved user opinions of the robot and several reports of fun interacting with Baxter. Notable positive comments included that one user “was surprised and impressed at how fast and fluid[ly] the robot was able to move” and another “liked how [Baxter] appears to get excited to play” when switching from the yellow neutral face to the purple happy face. The safety ratings of Baxter were also uniformly high, despite Baxter's occasional motion interpretation errors. Other strengths of this work are findings on the ability to influence how people think about working with Baxter via different leading and following roles. Users thought a lot about teamwork with Baxter during human-lead trials, sharing more comments about Baxter's performance, their own performance, and the hand-clapping teamwork. Experiences varied from easy (“I really liked how easily he learned my game”) to medium (“I may not have been the best teacher, but Baxter still learned a lot by round 3”) and even challenging/adverse situations (“the first time we were perfect, and that was super exciting. But once we did well, the mistakes in the next round were that much more devastating”). Nevertheless, users seemed to want to succeed in teaching Baxter, and some empathetic users even adjusted their motion sequence to fit Baxter's errors during the post-demonstration interactive play. In the broader social robotics picture, this experiment also provoked a number of complex emotional responses from people. Especially in the Block 4 interactions, users expressed joy at successes, and they also exhibited occasional cheeky responses to Baxter's errors. One non-technical user even talked to the robot, asking “Are you drunk, Baxter?” when the robot did a poor job reciprocating the demonstrated motion pattern.

The study design also had some shortcomings. Although the user behavior in this experiment was more naturally situated than in our previous spHRI work, the interaction could still be more natural; we required quite a bit of structured behavior from users to help Baxter interpret their motions. This requirement was especially problematic for users who were not adept at keeping a constant tempo. The chosen motion parsing and classification strategy further leads to a delay between when the user demonstrates each motion and when each move is classified. The system transparency could also be better. An additional robot thinking face while Baxter processes the participant motion data, for example, would help users understand the robot's state. Participants often recommended sound effects and experiment flow changes in the block surveys. Some wanted “a beat like the metronome from the teaching part” throughout their entire clapping experience with Baxter or a “clearer indication of [when] learning and playing phases start and stop, perhaps via audio” to help them focus their visual efforts on tracking Baxter's movement. Several users also requested a brief pause during robot-led conditions between Baxter's demonstration and the interactive human-robot play, perhaps inspired by the time the robot took to “think” about the demonstrated movements during the human-led trials. Furthermore, a few of the hand-clapping motions, especially DF and UF, were awkward for tall users. Our future research would benefit from automatically adjusting clap contact location based on user height.

Other drawbacks arose from the setting and the user population of the study. The experiment participant pool was fairly small and consisted mostly of young technical students. Within this group, we found that female users had a more positive impression of the robot than male users; this difference could the fact that most of our non-technical participants were also female. The study also took place in a lab setting that is different from future natural environments where humans and robots might interact. To ensure broader generalizability, we would need to run the experiment on a more diverse population in a less controlled everyday environment. The within-subjects design of the experiment may have exaggerated differences between conditions due to demand characteristics (Brown et al., 2011). We also must consider the fixed block ordering of the experiment when interpreting results and note the possible ordering effects on any condition differences. For example, participants might be more interested in the first block due to novelty effects and less engaged in the final block when the interaction has become more familiar. Users might also compare each subsequent block related to the previous experience, which is the same for each person in this study design. Hence, ratings might be better balanced in an experimental design with a varied trial ordering. A final challenge arising from the largely technical, robotics-savvy population of the experiment was that some people assumed that Baxter was using a vision algorithm to classify their motions. This belief is not inherently problematic, but it may have influenced the way people moved when demonstrating motions to Baxter, thus affecting Baxter's motion classification accuracy and attempted game pattern reciprocation. One user stated their belief in how the classifier worked explicitly, noting that there were “some mistakes during the training process, but [that] the accuracy was pretty good (considering [the algorithm] must differentiate between different hand poses quickly with the other hands somewhere in the background).”

### Key contributions and future work

Next research steps would involve trying to improve the robustness of Baxter's motion classification ability. The machine learning pipeline could be updated using the study data recordings of how people move and behave when in front of an actual robot. There may also be opportunities to improve user demonstration performance by offering advice on how to move during motion demonstrations, training additional bigrams, encouraging games that involve only bigrams of motion encapsulated in our original training and test datasets, and/or giving users a way to provide feedback to Baxter to enable reinforcement learning. Other improvement steps include adding more social feedback and auditory cues to the experiment, as suggested in user comments.

Overall, we are energized by signs of user fun and increasingly social opinions of Baxter over the course of the study. This work may be applicable to future HRI efforts on manipulating what users think about during interactions, considering how to get a person's attention, and designing future spHRI with appropriate cueing. The hand-clapping interaction itself may be a good way to help people learn how robots move and to break the ice when forming human-robot teams. Other future research directions from this bimanual clapping work include trying the sensing system on populations who are undergoing physical therapy for motor rehabilitation. Our findings, especially those on the classifier accuracy and social user responses to bimanual hand-clapping with a robot, can guide future spHRI research.

## Ethics statement

This study was carried out in accordance with the recommendations of the University of Pennsylvania IRB under protocols 822527 and 825490. The protocols were approved by the University of Pennsylvania IRB. All subjects gave written informed consent in accordance with the Declaration of Helsinki.

## Author contributions

NF was responsible for experiment preparation, data acquisition, data processing, and publication writing. KK advised throughout the experiment preparation, data acquisition, and data processing, and also supplied revisions for each publication draft.

### Conflict of interest statement

NF and KK have received research grants from the US National Science Foundation. KK has also received funding from the National Institutes of Health, Intuitive Surgical, Inc., IERION, Inc., Rolls Royce, Inc., the Wallace H. Coulter Foundation, the Defense Advanced Research Projects Agency, the Army Research Laboratory, Willow Garage, and the Pennsylvania Department of Health. NF now works at the University of Southern California. KK now works at the Max Planck Institute for Intelligent Systems. KK has also served as the Chief Scientist for Tactai, Inc. and VerroTouch Medical, Inc.
